# Supplementary figures and images for: Six1 promotes skeletal muscle thyroid hormone response through regulation of the MCT10 transporter
Source: Skelet Muscle. 2021 Nov 19;11:26. doi: 10.1186/s13395-021-00281-6 (PMC8607597; doi:10.1186/s13395-021-00281-6)

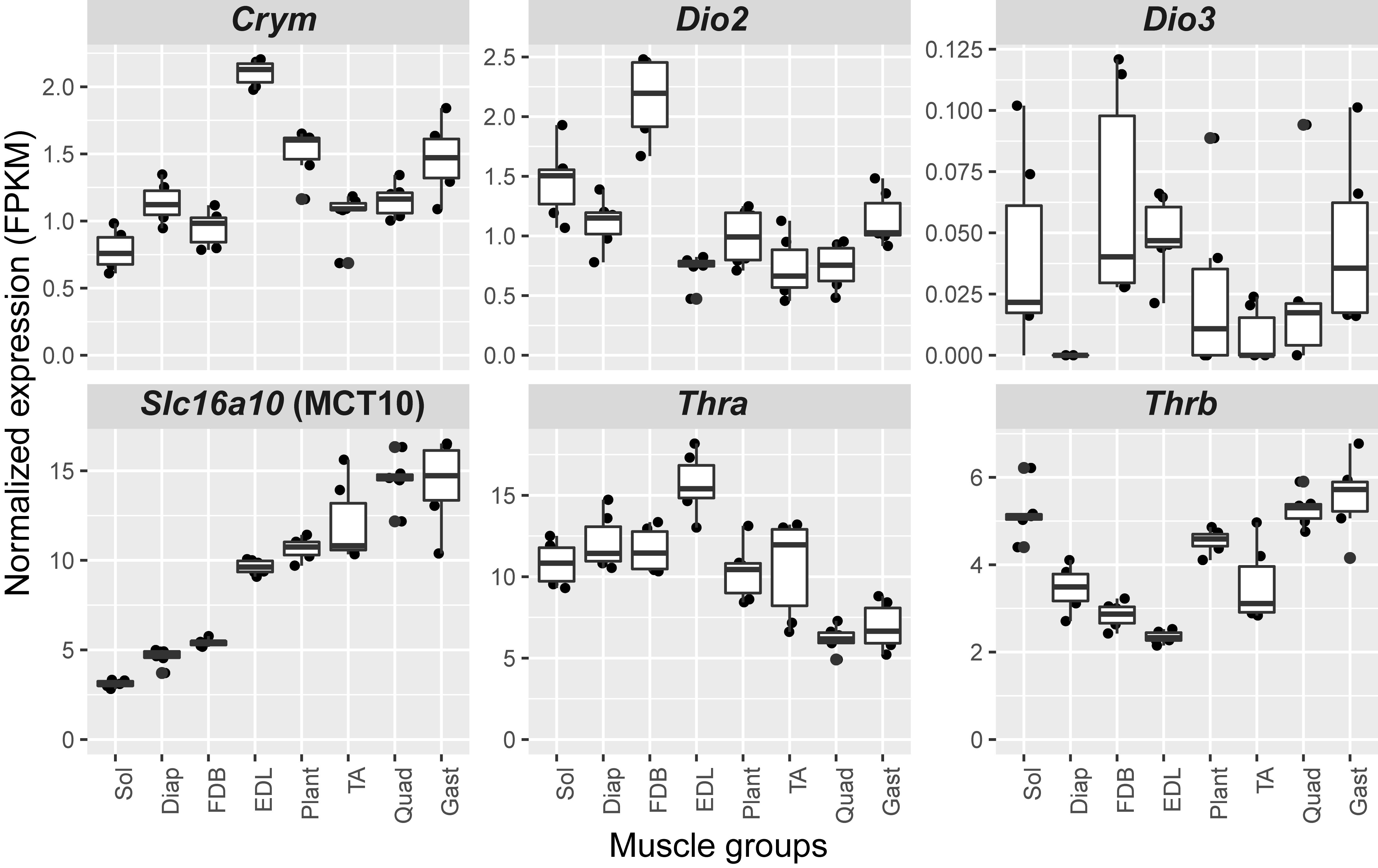

Supplement: Supplementary file 2 — Additional file 2: Figure S2. Expression of TH pathway genes in different muscle groups. The expression of the indicated genes was estimated from the muscleDB dataset and is represented as RPKM. Data analysis was performed as for Fig. 3. [file 13395_2021_281_MOESM2_ESM.tif]
